# Lectures on Fevers

**Published:** 1854-10

**Authors:** William Stokes

**Affiliations:** Regius Professor of Physic in the University of Dublin


Lectures on Fevers. By William Stokes, M. D., Regius Professor of Physic in the University of Dublin. *Lecture* IV.—It was my intention to have commenced to day the examination of the more important secondary diseases of fever; but we must postpone this part of the subject, and take the opportunity of studying the general symptoms of fever as they are presented, in their most aggravated and appalling form, in the case of the young man Hardcastle. This patient is a native of England, and has been but a short time in Dublin, and his case is full of instruction.

I have mentioned, that this patient is not a native of Ireland, because it is probable, that this circumstance has acted in modifying the symptoms of his disease. Had this patient been attacked with fever in his native place, he probably would have shown the symptoms of what is called typhoid fever, and the case would have been a comparatively mild one. But, at all events, this much we may believe, that different countries present endemic, sporadic, and even epidemic fevers, with characters in some degree peculiar to themselves; and we are not yet able to explain, why fever in one country differs so much from fever in another—why maculated typhus should be so common in Ireland, and so rare in England; nor why the fever in Paris should so constantly present a particular local lesion. Many causes, doubtless, act; and I suppose among others, such as climate, diet, soil, and so on, that the race and temperament are to be reckoned. And I have suggested that, if the various families of mankind have a physiological stamp, they, probably, have also their pathological peculiarities; however this may be, it becomes a curious subject of inquiry, to determine what are the modifications of the symptoms of the fever in Ireland or in any other country, when the patient is not a native of the country, and especially if he has not been long resident in it. The most extraordinary case I ever witnessed was that of a gentleman, a native of France, who, after a long exposure to contagion, contracted maculated typhus here. He was a man of a sanguineo-nervous temperament, and exhibited, during his illness, a succession of symptoms widely different from those which we commonly see in the spotted typhus of this country. He was profusely maculated, and the peculiarity of the case consisted in the irregular manifestations of various local symptoms, and the inconstancy of the general condition, especially with reference to excitement or collapse. This gentleman happily recovered, after passing through a storm of disease, such as I and his other attendants, Dr. O'Farrel and Dr. Graves, had never seen before.

Hardcastle is a young man who has been well educated, and he is evidently above the rank of the ordinary hospital patient. His symptoms have been, from an early period of the disease, alarming in the highest degree. You will seldom see a worse case of adynamic fever. We may safely say, that for the last fortnight, both by day and by night, there has been an uninterrupted struggle between medical art and the fell disease which is upon him. During that time he has been lying more like a decomposing corpse than a living man; but he has been kept from dying, by the bold and constant exhibition of stimulants, by tonics, and by food.

Some of you may wonder why it is that we thus go on with the increasing exhibition of stimulants—the untiring efforts to support life—although for the last ten days, at least, we have been hourly expecting his death? And the answer is, that we believe his disease to be one which may subside, sooner or later, under the law of periodic action. His recovery will then take place,—not in consequence of the action of any specific which has the power of curing fever, but from causes the nature of which is hidden from us. We are like the defenders of a post attacked by a powerful enemy, but yet expecting succor, and we seek to hold the place until that succor arrives.

There are two points in this case worthy of notice. One is, that the nervous system has been so little engaged. He has had occasionally a slight delirium, and latterly some agitation and slight subsultus tendinum; but, though he generally appears sunk in stupor, it is not true coma, but rather the stupor of exhaustion, for, when you rouse him, his intelligence appears to be good. Is this escape of the brain to be looked on as favorable, or the contrary? According to an old and well-founded opinion, all anomalous circumstances in fever are to be feared. You know the aphorism, "*Pulsus vultus et urina, bona, et æger moritur.*" This contains an important truth; but, in typhus, the want of nervous symptoms is in general favorable; for of the symptoms referrable to one of the three cavities, doubtless those indicating nervous lesion are the most formidable.

The second point to which I wish to direct your attention is, that, although this patient presents the symptoms of typhus so decidedly, he cannot be said to have any maculæ or petechiæ. In fact, his skin has been unaffected; there has been none of the so called exanthematous eruption of typhus; and, even at this advanced period, the existence of petechiæ is so doubtful, that I would not say that he has them. Yet, if ever a man had the *typhus gravior*, this man has it. His surface exhales the peculiar odor of typhus, strongly marked; he is prostrated to the last degree; he has been kept alive by the most powerful stimulation; he has a feeble heart, and well-marked secondary affections of the intestinal and bronchial surfaces; his mouth is full of sordes; his tongue black, dry, and cracked; his breath fetid. The stethoscope indicates a general and severe bronchial affection, and there is a wasting diarrhœa.

I am not going to take up your time by a discussion as to the distinctive characters of typhus and typhoid fever, about which so much has been written of late. But it would be, I think, difficult even for the advocates of the distinction, to declare in which class this case should be placed. In this instance, as in most others, the settlement of the question is of little value; for, whether the disease be typhus or typhoid fever, the treatment should be no other than that which we have so far carried out. I may say this much, however, that the presence or the absence of an early eruption having some similarity to an exanthem; or, again, the presence or absence of petechiæ, appears to be insufficient to justify our drawing a strong line of distinction between these diseases. As to the doctrine, that the early eruption in typhus is a true exanthem, and that its absence in any given case points out that the disease is not typhus, but typhoid fever, I would advise you to receive it with more than caution. Again, it is stated, that the non-maculated fevers are often protracted and dangerous; so they are, but not so often as they are short and easily managed. And I have not seen that relief of organs in typhus, which some describe as the rule, when the eruption appears. How, then, are we to look on the eruption? Certainly not as a distinctive mark between two different diseases. Gentlemen, we know that typhus fever will run its course, and destroy life, without the production of any hitherto ascertained anatomical change. We know further, that in many cases, local alterations are formed; but that there is the greatest inconstancy in different epidemics, and in different patients during the same epidemic, in the seat, amount, in the time of appearance, and in the results of these local changes. The cutaneous affection is plainly one of this group of secondary affections; and its absence or presence can no more be said to distinguish the disease, than the absence or presence of any other of the secondary affections. I think we may admit, that an early and florid eruption is often met with in cases in other respects well marked; and again, that such cases generally require and bear stimulation.

To return to the case of Hardcastle. We observe that there has been as yet no tendency either to the critical or periodical retrocession of the secondary diseases, nor to the critical or periodical termination of the general affection. The fourteenth day passed by without any crisis or without any subsidence of the disease; the eighteenth day passed by; the twenty-first has passed by; and he is now in the twenty-fourth day of this terrible disease, kept alive by the use of wine, administered every hour, and also by the occasional free use of hot brandy punch. He presents an appearance to-day to which I wish specially to direct your attention. You may remember that Saturday last was the twenty-first day, and it was then reported to me that the patient was getting a bed-sore. Upon examining him, we found, in the ordinary situation of bed-sores, a blush of redness and a slight degree of œdema; and beyond that there was nothing remarkable. There was this point, however, which I observed at the time although it was not until to-day that I thought it of importance, namely, that there was already a solution of continuity of the skin. Now, this is remarkable; and if such a case should occur to me again it would awaken my attention. In the ordinary cases of bed-sores, we seldom see a solution of continuity of the skin in an early period; we see lividity, blackness if you will; but a solution of continuity of the skin before the ordinary appearances of mortification have occurred, is extremely rare.

In this patient, on Saturday, just at the very centre of the livid patch on the skin, there was a slight solution of continuity. During the evening, the nurse observed that several dark-colored vesicles or pustules were making their appearance on various parts of his body. Mr. Parr saw these on Sunday, and he found that between the shoulders there was an eruption of livid tumors, which appeared to be something between vesicles and pustules; and to-day (Tuesday) such of you as saw the patient will not soon forget the extraordinary appearance of his back. I have been attending on fever in this Hospital since 1827, and I never saw anything of this kind before. Now, it is a very remarkable circumstance, that this eruption of gangrenous vesicles or pustules should have occurred upon the twenty-first day; and this case, as far as it goes, appears to strengthen an opinion which I have long held, that we were in error in attributing what are termed bed-sores in fever, simply to mechanical causes. The general idea is, you know, that they are simply the result of pressure long continued on a particular part of the body, from the neglect of turning the patient in bed, of pressure combined with the effects of position. Now, there can be no doubt that these are predisposing causes, but whether they are the sole and entire causes is another matter; and I am, to a great degree, convinced, that the bed-sore in fever is often one of the group of secondary affections analogous to the ulceration, or to the tumefaction and ulceration, of the glands of the intestine—analogous to the bronchial affection which occurs in the middle stages of the disease,—analogous to the other secondary organic diseases of fever. And if analogous to them, it should be more or less observed to be under the law of periodicity; it should appear at a certain time, and it should disappear at a certain time, or, at least, exhibit a tendency to do so. We have observed this very curious fact. There are cases in which the system seems to be in a state that bed sores will form from the slightest possible causes, that is to say, that whenever there is any irritation or any pressure, no matter how slight, a bed-sore will form in any part of the body. Some years ago we had a patient in this hospital, who, after the twelfth day, showed this tendency to bed sores. She had bed sores on the nates, and one on each shoulder. Then the tendencv to the multiplication of these bed-sores increased, and every morning two or three new local diseases of this kind were discovered. Wherever there was the slightest possible pressure, we found a gangrenous spot; in the fold of the arm, in the fold of the pectoral muscle, where the mamma leaned upon the arm, where the head leaned upon the hand in sleep, the mark of a black hand was stamped upon the face,—where one leg lay on the other,—in every possible position in which anything like pressure was made, or irritation excited, there were bed-sores; and this went on repeating itself, day after day, up to a certain day, until there were about thirty sloughs in different parts of the body; and from that day,—I might say from an hour of that day,—no more bed-sores formed, and the patient then had to go through the process of throwing off all those sloughs the granulation of the cavities from below, and their cicatrization. She was kept lying on her face for upwards of a month, and finally recovered.

Now, what I want to draw your attention to in the case of Hardcastle is, that this extraordinary eruption of gangrenous patches on his back is not of the nature of bed-sores in the ordinary sense of the word. These patches are not produced by pressure. We find them in great abundance in the hollow of the back, where pressure is relieved by the pelvis and by the shoulders. We find them in abundance in the interscapular region; but if we wanted additional proof, it is this,—that we find them on the anterior portion of the thorax. They came out as vesicles. These vesicles became hard at the top, then black, and soon the mass dropped out; and this patient's back in now as if you took a sharp gouge and punched out circular portions of the flesh in a vast number of spots. Now, it can be hardly doubted, that this singular appearance is simply an example of a secondary disease affecting the surface; and it is very remarkable that it should have appeared on one of the critical days—upon the day when, in the ordinary course of fever, the disease should have subsided. And it strengthens greatly the opinion, that not only the general disease is under the law of periodicity, but that the secondary alterations are so too.

As a general rule, gentlemen, in prognosis, the occurrence of any vesicular eruption whatsoever in fever is bad. We have in fever several forms of vesicular eruptions. But, suppose a patient in the eighth or tenth day of fever, when he is otherwise going on well, you take his hand to feel his pulse, and you are surprised at seeing a vesicle upon his arm. Do not neglect that—do not overlook it. The mere circumstance of that solitary vesicle forming so silently, without any pain, without any notice, points out that there is mischief before you—that the case is likely to go wrong.

It is a very important prognostic. Here we have on this patient an eruption of vesicles running rapidly to deep gangrenous destruction of the part, for some of the cavities formed by them are singularly deep, though circumscribed. Now, suppose that in place of that disease attacking the skin, upon the fourteenth day, on the tenth day, or whatever day you please, in any case, a similar condition should attack the intestine. Suppose that the typhoid matter suddenly infiltrated a gland in the intestine, and that that should fall as suddenly to gangrene, and that there was a solution of continuity; you will at once see the progress of the worst form of the typhoid secondary disease of the intestine. You may look on this man as at this moment turned inside out, and that on the surface of his skin you are able to learn the history of the worst form of the typhoid ulceration of the intestine. This extraordinary condition of his surface no one will say is inflammation of the intestine? Certainly not. Whether this man is to live, gentlemen, or to die, I believe none of us can venture to say. Of course, the chances are enormously against him; but, in the treatment of fever, you are never to despair so long as the patient can swallow. So long as he is able to take nourishment, so long as he is able to swallow wine, no matter how dreadful or apparently hopeless the symptoms may be, you are not to desert him, but—to use the phrase of our glorious sailors--you are to fight the ship while she swims. In a disease under the mysterious law of periodicity, every hour of compelled life is a clear gain. And, over and over again, you will find that your efforts will be crowned with success. You will see a patient lying with his back icy cold; you will see him pulseless,—his lungs filled up with secretion,—his belly tympanitic,—with dreadful diarrhœa,—the lower extremities gangrenous,—himself in a state of insensibility,—and yet, even under those circumstances, a recovery is possible. But that recovery can only be effected by the steadfast determination of the physician not to desert his post until the vital spark has actually fled; and, if you commit an error in holding on,—in hoping against hope,—at all events it is an error on the right side.

---

*Lecture* V.—With reference to the case of Hardcastle, with which we were occupied at our last lecture, we may hope that the symptoms have at last yielded. The great interest of this case consists in its having been an example of a fever in which the patient was scarcely maculated yet in which the stimulating treatment had to be pursued with an activity as great or greater than that which we are called on to employ in the worst cases of spotted typhus.

On looking over the notes of the case, I find that on his admission, which was on or about the seventh day, he had a few scattered maculæ on the abdomen, of a large size, and of a leaden gray color. He then had diarrhœa, abdominal tenderness, and ileo-cœcal gurgling; and the sounds of the heart, though weak, preserved their natural mutual relations as to force and duration. Doubtless the patient at this period might have been well described as laboring under typhoid and not typhus fever, according to the distinctions now in vogue; but on the fourteenth, or the twenty-first, or the twenty-fourth day, he would be a bold man who would declare that the case was not typhus of the worst description. He is now at the twenty-eighth or thirtieth day of his illness, and we have every reason to believe that all will now go well. Since the eruption of gangrenous vesicles of pustules, which occurred on the twenty-first day, there have been no new appearances of this form of disease, there has been no new bedsore, nor have the gangrenous patches spread as might have been expected; two of them are in the form of sinuous cavities, but even these show signs of healing. These sores on the back were treated first, you will remember, by the simple and afterwards by the fermenting poultice; but as the latter gave considerable pain, the nurse returned to the simple poulticing, and now, under the advice of my colleague, Mr. Smyly, we have changed our plan, and are using stimulating dressings to the ulcerated surfaces. In the management of these sores, whether they be the ordinary bed-sores or examples of the gangrenous pustules, both of which appear to be of the nature of the genuine secondary affections of typhus under the law of periodicity, it sometimes happens that we have to deal with extensive sinuses. I have seen them sometimes of not less than seven inches in length. In most cases, the best treatment for them will be stimulating injections, using as an apparatus the vulcanized india-rubber bottle with a long and slender ivory pipe; and it is generally requisite at first to wash out the sinus with tepid water, and afterwards to inject some of the metallic solutions—diluted solutions of sulphate of copper, nitrate of silver, or sulphate of zinc—and when the discharge is very fetid, you may use the decoction of bark, or the solutions of chloride of lime or soda. An excellent dressing when the sore is open is the Canada balsam combined with oil, or a mixture of equal parts of castor-oil and balsam of copaiba. You will derive advantage, also, from the employment of pressure by means of flat compresses of lint, and a roller when it is possible to apply it, or in other cases you may employ strapping with adhesive plaster. When the sinus is in a depending position, and matter accumulates in its lower portions, it is sometimes necessary to make a counter opening; but this operation should rather be delayed until the system of the patient has improved; the case is then to be treated as an ordinary surgical case.

Now let me draw your attention to the diligence with which the administration of wine has been pursued in this case. We began the exhibition of wine on the 2nd of November, which was the eighth day of his disease; and, for the next four days, the quantity administered was six ounces daily. The wine used all through was port, of an excellent quality. From the 6th to the 11th he had twelve ounces daily, and from the 11th to the 18th his daily allowance was twenty-four ounces. During the next three days it was reduced to eighteen ounces; and, from the 21st to the 26th, to twelve ounces per diem. On the 26th and succeeding days he had ten ounces; on the 28th the quantity was reduced to eight; on the 4th of December he had but four ounces; and the wine was omitted altogether on the 5th. Besides all this, a tumbler of hot brandy punch, containing two ounces of the best brandy, was administered from time to time whenever the patient's state seemed to require it; and in this way he used about twenty-four ounces of brandy, and four hundred and thirty-eight ounces of wine, and, in addition, bark, musk, and ammonia were freely exhibited.

Some may suppose that this quantity of wine was excessive. Such is not my opinion, nor will it be yours when you come to treat the typhus of this country in a large hospital. I am sure we might have given him with safety much more, and I doubt if we could have done with less. And the result of the case confirms what I have said to you before, that in a case of fever, no matter how terrible the group of symptoms may be, you are not to despair so long as the patient can swallow. Bear in mind, too, that all through, Hardcastle has been carefully fed with chicken broth, beef-tea, and jelly; and we have now passed that period when, under the law of periodicity, his terrible disease will subside.

This case brings to my recollection two remarkable instances in which an extremely bold use of stimulants was the means of saving life. Both were cases of most malignant typhus, and in both instances the patients were medical men. In the first, we administered more than two dozen of wine, of which six bottles were champagne; and besides this, the patient had not less than six bottles of brandy. His fever did not pass the twenty-first day; it was, therefore, at least ten days shorter than that of Hardcastle's, so that the stimulants were not only in greater quantity, but exhibited within shorter intervals of time. The symptoms in this case were the most extreme adynamia, continued coma, persistent boldness of the breath, enormous vibices, and a general purplish black color of the skin. We could discover no local lesion; the recovery was perfect, and the gentleman has now remained for many years in excellent health. He was a man of very temperate habits. In the second case, on about the twelfth day of the fever, I was called to see the patient; he had got no stimulants. The nervous system was free, the surface was cold, and the pulse almost imperceptible; the integuments of the lower extremities, from the middle of the calf downwards, were black, and hung in loose wrinkles on the limbs. This patient swallowed about sixteen ounces of brandy in the course of a few hours; reaction was established, and the case did well. We expected extensive mortification in the legs, but within four-and-twenty hours from the commencement of the exhibition of stimulants we found that the circulation was fully re-established, the extensive blackness had disappeared, the limbs had recovered their fulness, and the subsequent loss of integument, which occurred only in the feet, was very trifling. In this case there was a petechial eruption, approaching to purpura; but although it is generally true that stimulants are more often called for in the maculated than in the non-maculated cases of fever in this country, yet you must bear in mind, that as in the case of Hardcastle, you will meet with a typhoid fever, if you choose to call it so, in which the most energetic stimulation is required. I have given an example of this in my paper on the state of the heart in typhus fever, in vol. xv. of the *Dublin Medical Journal* (first series). The patient used 292 ounces of wine, 20 ounces of brandy, 7 bottles of porter, and had besides various medicinal stimuli and abundance of food. The principal symptoms were extraordinary prostration, coldness of the surface, and feebleness and irregularity of the heart's action; and it was not until the end of the eighth day of the exhibition of stimulants in great quantities that any favorable influence was produced in the circulation; and the case strongly illustrates the advantage of persisting in the use of stimulants, although for days together no amendment seems to follow their employment.

I have sometimes observed that students were under a misapprehension about the doctrines which we have long held in the hospital with respect to the condition of the heart as a guide for the use of wine. They have come to the erroneous opinion that we are only to give wine where we find the want of the first sound of the heart, and that we are not to give wine where the heart is acting well. This is a mistaken view of the matter. What we have established as to the state of the heart in connection with the effect of stimulants, is simply this,—we have ascertained that the efficacy of stimulants is often directly as the debility of the heart. It has been also ascertained that the power of bearing stimulants, their effect upon the nervous system, their good effects on the general condition, are directly as the weakness of the heart. We may lay down as a rule, that there are three conditions of the heart to be looked at by the practical man in the treatment of fever. In one we have an excited heart—a violently excited heart all through the case; and this heart may be excited and violent, although the symptoms be those of extreme adynamia, although the surface be cold, the breath cold, and the pulse so feeble that it cannot be discovered. Nay, the heart may act with great force for several days, and yet there be no pulse at the wrist. This is one case. In the next case, we find exactly an opposite condition, in which the systolic force of the heart is diminished. This is shown by loss of impulse of the heart, by diminution of the first sound, and, in certain cases, by extinction of the first sound of the heart while the second remains. This is a case which calls for wine, and in which you should give it; it is a case in which in the vast majority of instances, wine will agree with the patient. There is a third set of cases in which the heart does not seem to be implicated at all in the course of the disease, in which, notwithstanding the existence of the most extraordinary group of symptoms affecting various organs, the heart, in the middle of the storm, seems to be in a state of calm and quiet. If we compare these three sets of causes with a view to prognosis, we may arrange them in this way. The case of excited heart all through, with feeble pulse and with adynamia, is unquestionably the worst case. There is no worse symptom in fever than an excited heart. It is especially a bad symptom when, with that excitement, we find a feeble pulse. The next will be the case of sinking of the heart; and the most favorable case is that in which, as I said before, the heart seems to escape disease. But you are not to suppose, that because you have an excited heart you are not to give wine if the symptoms of the patient require it; and you are not to suppose that, because the heart is not affected at all, you are to withhold wine if the general symptoms of the patient require it. You are not to found your exhibition of wine or stimulants upon any one thing; you are to take the general state of the patient into consideration. What we have done, is to discover an intelligible practical rule which will guide you in the use of wine in certain, I think in many cases; but you are not to suppose that because this man has a clear first sound at his heart, therefore you are not to give wine. You are not to suppose that because the heart is safe you can do without wine. Now, in the case of Hardcastle, recollect that although his heart seemed to escape, or was at most only feeble through the course of the disease, what frightful adynamia existed; how for day after day the patient's face was hippocratic, or almost so; how the general character of the disease was that of the most terrible putrescent fever; yet his heart escaped. And here is the result. We have given that man upwards of twenty bottles of wine and twenty-four ounces of brandy and now, on the twenty-eighth or thirtieth day of the disease, we have the satisfaction of feeling that his case may be set down as among the triumphs of medicine. I wish also strongly to impress on you the great importance of the use of other forms of nourishment in this disease; for we must not only keep up the nervous energy of the system by wine, but we must support nature by food. There is no mistake more fatal in fever than the withholding of food. I was early taught the importance of the use of careful nourishment in fever by my friend and colleague, Dr. Graves. I remember once, Dr. Graves, when speaking of the necessity of the use of nourishment in fever, made use of these words,—"If you are at a loss for an epitaph to be placed on my tomb, here is one for you,—'*He fed fevers.*'" In addition to the prejudices with which the inflammatory doctrine imbued so many minds, with respect to the use of food in fever, there was a new set of arguments raised against it, in consequence of the experiments of an American physician. I allude to the case observed by Dr. Beaumont, and so often quoted since. In this remarkable case, various medicinal substances and articles of food were introduced through an external fistula into the stomach, their effects being noted, as also the conditions of temperature, vascularity, etc. A set of results were subsequently published in connection with the action of the stomach upon food. One of the results stated to have been thus obtained was, that the existence of the state of fever altogether suspended the process of digestion. Here was a statement which had the appearance of being the result of strict observation. It influenced a number of young men; but did it influence those who had once been in charge of a fever hospital? Not at all; because those men knew very well that, no matter what Beaumont might say about the stomach not digesting when the patient had fever, in thousands of cases patients in fever digested remarkably well, required food, and derived benefit from it. In a large number of cases of typhus fever, the stomach has an excellent power of digestion; and, I believe, if we were bold enough, we would find that many articles of food usually forbidden to fever patients might be given to them with safety. A curious incident was related to me which shows that the stomach in fever is capable of digesting even a rather coarse article of food. A lady who had been recently married was attacked with extremely severe petechial fever; she was covered with dark-colored maculæ, and the disease had run to about the twelfth or thirteenth day. She was attended by several eminent physicians. Her case was an extremely bad one, and her life was all but despaired of. She was violently delirious. Her husband had occasion to leave the house on some business. At the period of the dinner hour of the family, the servants were cooking a rump of beef and cabbage, and the odour of it filled the house. In her delirium, she called for some of the beef and cabbage; she was then, you must understand, in severe fever, and covered with maculæ. Her sister, who was then attending her, believing she was dying, thought it only right to indulge her, from the feeling that it was right to indulge the request of a dying person. She proceeded to the kitchen, and as soon as the beef was boiled, cut a very large mass of beef and cabbage; and this was brought up smoking hot to the lady's bedside, when she devoured it with great avidity. Shortly afterwards her husband came in, and was told what had happened. He became terrified, and sent for physicians in every direction. Four or five assembled; time was pressing, and every one agreed that something should be done. At length the late Dr. Harvey, a practical physician of the very first class, arrived. He was laid hold of by the agonised husband, forced up stairs, and his opinion earnestly requested. At that time, the stomach pump was not in fashion, but every one agreed that something decisive should be done, that an emetic should be given, or some extraordinary effort made to get this mess of beef and cabbage out of the lady's stomach. When Dr. Harvey went to the bedside, he found the patient in a tranquil sleep. He turned round, and when anxiously appealed to what should be done, he said—"You had better wait till she wakens; let her sleep it out." She slept for four or five hours; awoke wonderfully better, and on the following day was out of danger. I do not give you this case to induce you to feed your patients with salt beef and cabbage in fever; but it is very important, as showing that in typhus fever, with maculæ, the stomach is capable not only of digesting such a coarse article of food as salt beef, but that even such food may have a good effect.

